# Socioeconomic, Lifestyle and Dietary Factors Associated with Dietary Supplement Use during Pregnancy

**DOI:** 10.1371/journal.pone.0070733

**Published:** 2013-08-13

**Authors:** Camille Pouchieu, Rachel Lévy, Céline Faure, Valentina A. Andreeva, Pilar Galan, Serge Hercberg, Mathilde Touvier

**Affiliations:** 1 Nutritional Epidemiology Research Team, Sorbonne Paris Cité Research Center, Inserm U557, Inra, Cnam, Paris 13 University, SMBH Paris 13, Bobigny, France; 2 Reproductive Biology Unit-CECOS, Jean Verdier Hospital, Bondy, France; 3 Public Health Department, Avicenne Hospital, Bobigny, France; Baylor College of Medicine, United States of America

## Abstract

**Background:**

Information on dietary supplement (DS) use during pregnancy is largely lacking. Besides, little is known about the share of DS use as self-medication versus such use following a physician's advice/prescription. Our aim was to evaluate DS use and its socioeconomic, lifestyle and dietary correlates among pregnant women participating in the French NutriNet-Santé cohort study.

**Method:**

Data were collected by self-administered web-based questionnaires. Food intake was assessed by repeated 24 h dietary records. 903 pregnant women provided data on their DS use (both “regular” DS and medication containing mainly vitamins/minerals). Supplement users were compared to non-users by unconditional logistic regression.

**Results:**

DS use—in general and as regards folic acid in particular—was positively correlated with age, being primiparous, having higher income and belonging to a higher socioprofessional category. DS users had significantly higher dietary intakes of most vitamins and minerals. The proportion of DS users (e.g., those reporting use at least three days a week) increased significantly with the trimester of pregnancy (58.0%, 62.2% and 74.5%, respectively). 50.2% of women in their 1st trimester used folic acid. The proportion of iron users tripled from the 1st to the 3rd trimester (18.5 to 63.9%). DS use was prescribed or recommended by a physician in 86.7% of the cases.

**Conclusion:**

This study provided new and detailed information on DS use and its correlates during pregnancy. Even in this relatively well-educated population, folic acid supplementation at the beginning of pregnancy was inadequate and was associated with socioeconomic and demographic disparities.

## Introduction

Pregnancy is a physiologically-specific period during which the needs for some nutrients increase. While the nutrient requirements of the general population can be met by an adequate diet, risk of suboptimal micronutrient intake is common during pregnancy [1] and supplements might be useful to meet dietary requirements for specific key nutrients. In France as in several other countries, 0.4 mg/day of folic acid is recommended before the conception, at the beginning of pregnancy to reduce the risk of neural tube defects (NTD), and also later during pregnancy, to prevent megaloblastic anemia in the mother [2]–[7]. The 2011 incidence of NTD in France was estimated at 1.0 case per 1000 births [8], while upwards of 200 cases per year could be prevented by improving folic acid intake [9]. This has led some countries to proceed with large-scale dietary folic acid fortification programmes [10]. The prevalence of anemia in developed countries (due to iron deficiency in 2/3 of the cases) is estimated at around 5% before pregnancy and is tripled during pregnancy [11]. Anemia is a risk factor for preterm delivery, low birth weight and cognitive impairment in the infant [12], [13]. Vitamin D deficiency has been associated with the risk of pre-eclampsia, gestational diabetes, preterm delivery, low birth weight, and low fetal bone mineral content [14]. Iodine deficiency is associated with maternal and fetal goiter, cretinism, intellectual impairments, neonatal hypothyroidism, increased risk of miscarriage and infant mortality [15]. Thus, folic acid, iron, vitamin D and iodine supplementation at moderate doses is recommended for pregnant women at risk of deficiency. In contrast, other systematic supplementation is not necessary in the absence of specific pathological situations and some supplements should even be avoided [16]. Excessive intake of retinol [17] or vitamin E [18], for example, may have serious deleterious effects on fetal development. According to the Developmental Origins of Health and Disease (DOHaD) concept [19], exposure to nutritional factors in utero is likely to have major health consequences in later life, notably through epigenetic mechanisms [20], [21].

Despite being of major public health concern, little information is available regarding dietary supplement (DS) use in pregnant women worldwide [4], [22]–[26]. Some studies conducted in various countries have suggested that DS use may be more frequent in older [24], [27], [28], well-educated [24], [25], [27], [28], non-smoking and primiparous women [24]. However, information is lacking about differences in nutrient intake from food between pregnant DS users and non-users. If DS use is indeed associated with different sociodemographic, economic, lifestyle, and dietary factors, an accurate assessment of these associations is necessary in order to better target the nutritional recommendations regarding pregnant women's supplementation with specific nutrients.

Our objectives were: 1) to investigate the demographic, socioeconomic, lifestyle and dietary correlates of overall and folic acid DS use in pregnant women included in the NutriNet-Santé cohort study; and 2) to assess the role of physicians in the motives for DS purchases and the proportion of self-medicating users.

## Methods

### Study population

Worldwide, the NutriNet-Santé study is the first large-scale population-based prospective cohort study that is exclusively Internet-based. It aims at elucidating the relationships between nutrition and chronic disease risk, as well as defining the determinants of eating behaviour [29]. It was launched in France in May 2009. Adults (≥18 y) living in France and having access to the Internet are recruited via mass-media campaigns. The study was approved by the Institutional Review Board of the French Institute for Health and Medical Research (IRB Inserm n°0000388FWA00005831) and the “Commission Nationale de l'Informatique et des Libertés” (CNIL n°908450 and n°909216). Written informed consent was obtained from all subjects.

### Data collection

Participants fill in self-administered web-based questionnaires at baseline and then regularly during the follow-up. Several of the baseline questionnaires were tested and compared against traditional assessment methods (paper questionnaires or interviews by a dietitian) [30]–[32]. In the NutriNet-Santé study, 60388 women had completed the DS questionnaire.

#### Pregnancy data

At baseline and regularly over the follow-up, data on current pregnancy and due date were collected through health status questionnaires.

#### Dietary supplement use

The questionnaire regarding DS use was administered two months after baseline in the entire cohort. In our study, we considered as DS both regular DS and medicinal supplements mainly composed of vitamins and minerals, which are treated as pharmaceutical products in France. Participants were asked if they were currently taking any supplement at least 3 days a week. They were also asked to specify the type of DS using a list of 34 different nutrients and substances. They had to refer to the nutritional information on the packaging of the DS that they were currently taking. Circumstances of DS purchase (notably role of physicians versus self-medication) were also reported.

#### Demographic, socioeconomic, lifestyle and behavioural data

At baseline, self-administered questionnaires were used to collect data on demographic, socioeconomic and lifestyle characteristics, including age, geographical region, marital status, number of children, educational level, socioprofessional category, and income.

#### Dietary data

Each year, the participants are asked to complete three non-consecutive self-administered web-based 24 h dietary records, the days for which are randomly assigned during a two-week period (two days during the week and one day during the weekend). All foods and beverages consumed at breakfast, lunch, dinner and at all other occasions are recorded. For foods with potentially high nutrient variability, participants are also asked to provide the brand name. The participants are asked to estimate the portion size for each reported food and beverage item using validated photographs [33]. Daily dietary intakes of energy and nutrients are then calculated using the NutriNet-Santé food composition table, which includes more than 2500 different foods.

Knowledge of official nutritional recommendations as provided in the French National Nutrition and Health Programme (PNNS) was also assessed. Finally, a specific questionnaire was used to assess the opinion and behaviour of women as regards organic food.

### Statistical analyses

All women who entered the cohort before September 2012 and had completed the DS questionnaire while pregnant were included in the present study (n = 903).

We estimated the proportion of DS users overall and according to the trimester of pregnancy. Types of DS as well as circumstances of purchase were also described.

DS users (overall and specifically regarding folic acid) and non-users were compared by age-adjusted unconditional logistic regression analyses, regarding their sociodemographic characteristics (age, geographical region, marital status, number of children, education, income, and socioprofessional category), knowledge of official nutritional recommendations and organic food consumption. Odds ratios (OR) and 95% confidence intervals (CI) were calculated.

The mean daily intake of dietary micro- and macro-nutrients was compared via logistic regression between DS users and non-users after adjustment for age, number of 24 h records and energy intake. Only women who provided at least one dietary record during their pregnancy and who were normo-energy reporters according to the Goldberg criteria [34] were included in this part of the analysis.

A P-value<0.05 was considered significant in all statistical tests. All tests were two-sided. Analyses were carried out with SAS software (Release 9.1, SAS Institute Inc., Cary, NC, USA).

## Results

Among the 903 pregnant women included in this study, 31% were in the 1^st^ trimester of pregnancy, 36% in the 2^nd^, and 33% in the 3^rd^ at the time of the DS questionnaire completion. Sociodemographic characteristics of the study population are presented in Table 1. The average age of the participants was 31.7±4.07 years. A high proportion of pregnant women (64.9%) used DS at least three days a week. The corresponding proportion among the non-pregnant women of childbearing age in the cohort was only 29.1% (data not shown). The proportion of DS users increased significantly with the trimester of pregnancy (58.0%, 62.2% and 74.5% in the first, second and third trimester, respectively).

**Table 1:** Demographic, socioeconomic and lifestyle correlates of dietary supplement use in pregnant women of the NutriNet-Santé cohort study.

	All Pregnant		Supplement		Supplement		Age adjusted logistic	
	women		Non-Users		Users^1^		regression analyses	
	(n = 903)		(n = 317)		(n = 586)			
	n	%	n	%	n	%	OR	P^2^
Age^3^	31.7	4.07	31.1	4.22	32.1	3.94	1.06 [1.02–1.10]	0.001
Geographical region								0.0007
Paris metropolitan area	198	21.9	46	14.5	152	25.9	1.00	
North	66	7.3	33	10.4	33	5.6	0.26 [0.09–0.77]	
North-West	142	15.7	48	15.1	94	16.0	0.61 [0.38–0.99]	
Center	225	24.9	76	24.0	149	25.4	0.61 [0.40–0.95]	
South-West	71	7.9	27	8.5	44	7.5	0.52 [0.29–0.93]	
North-East	97	10.7	37	11.7	60	10.2	0.52 [0.31–0.89]	
South-East	89	9.9	42	13.2	47	8.0	0.35 [0.20–0.59]	
Corsica & overseas depts/territories	15	1.7	8	2.5	7	1.2	0.26 [0.09–0.77]	
Marital Status								0.6
Married or living with partner	847	93.8	295	93.1	552	94.2	1.00	
Single	56	6.2	22	6.9	34	5.8	0.84 [0.48–1.48]	
Number of children								
0	518	57.4	171	53.9	347	59.2	1.00	0.003
1	262	29.0	97	30.6	165	28.2	0.70 [0.50–0.97]	
2 & more	123	13.6	49	15.5	74	12.6	0.52 [0.34–0.82]	
Education								0.4
<12 years of schooling	37	4.1	16	5.0	21	3.6	1.00	
> = 12 years of schooling	866	95.9	301	95.0	565	96.4	1.32 [0.67–2.60]	
Income (€/month)								
<1670	71	7.9	35	11.0	36	6.1	1.00	0.0004
1670–3130	328	36.3	134	42.3	194	33.1	1.35 [0.81–2.27]	
>3130	504	55.8	148	46.7	356	60.8	2.09 [1.25–3.50]	
Socioprofessional category								0.008
Executive and intellectual professions	322	35.7	94	29.7	228	38.9	1.00	
Intermediate professions	253	28.0	79	24.9	174	29.7	0.96 [0.67–1.39]	
Employees	280	31.0	119	37.5	161	27.5	0.61 [0.43–0.87]	
Manual workers, farmers and self-employed	28	3.1	14	4.4	14	2.4	0.42 [0.19–0.92]	
Never employed	20	2.2	11	3.5	9	1.5	0.45 [0.17–1.16]	
Knowledge of official nutritional recommendations^4^								0.1
Poor (0–2)	191	21.2	78	22.9	113	19.0	1.00	
Average (3)	217	24.0	73	23.2	144	25.1	1.35 [0.90–2.02]	
Good (4–5)	495	54.8	166	53.9	329	55.9	1.34 [0.94–1.89]	
Organic food consumption^5^								0.2
Never (avoid organic products)	191	25.5	79	29.7	112	23.2	1.00	
Indifferent to organic food	88	11.7	32	12.0	56	11.6	1.18 [0.70–2.00]	
Occasional consumption	239	31.9	75	28.2	164	34.0	1.53 [1.03–2.28]	
Regular consumption	231	30.8	80	30.1	151	31.3	1.37 [0.92–2.05]	

1 Dietary supplement users were defined as the subjects who used dietary supplement(s) at least 3 days a week at the time of the DS questionnaire.

2 P for linear trend (with adjustment for age, number of children, income, and knowledge of nutritional recommendations) or overall P (for all other variables).

3 Values are n % for all variables except for age where values are mean SD.

4 From the French National Nutrition and Health Programme.

5 Determined by multiple correspondence analysis of data from a questionnaire on organic food consumption (5 clusters defined by the first 3 discriminant axes). Because of missing values, the proportions of subjects were calculated with 483 supplement users and 266 non-users.

### Demographic, socioeconomic and behavioural correlates of overall and folic acid dietary supplement use

As compared with non-users (Table 1), pregnant women who used DS were more likely to be older, to live in the Paris metropolitan area, to have had no biological children, to have a higher income, and to occupy an executive/high-skilled position (compared to manual workers and low-skilled staff). The same correlates were statistically significant specifically for folic acid supplement use (P = 0.02 for age, 0.01 for geographical region, 0.0001 for number of children, 0.001 for income, and 0.007 for socioprofessional category, data not tabulated).

### Overall and specific DS use according to the trimester of pregnancy

The three substances most commonly reported were folic acid, iron and magnesium (Table 2). About half of the women used folic acid in the 1^st^ trimester. The proportion of iron users tripled from the 1^st^ to the 3^rd^ trimester. 15.5% of women reported taking vitamin D supplements on a regular/daily basis (information about single-dose use was not available). The proportion of vitamin D users during pregnancy was respectively 16.9%, 14.8%, 15.4% and 9.8% in women who delivered during the spring, summer, fall and winter, respectively (data not shown). The proportion of pregnant women using iodine DS reached 25.6% in the 2^nd^ trimester. Retinol supplement use reached 5.8% during the last trimester of pregnancy. Vitamin E supplements were used by 29.0% of women in the 2^nd^ trimester. About 11% of the women reported taking herbal supplements.

**Table 2:** Overall and specific dietary supplement use in pregnant women of the NutriNet-Santé cohort study, according to the trimester of pregnancy^1^.

	All pregnant		1^st^		2nd		3^rd^		
	women		trimester		trimester		trimester		
	(n = 903)		(n = 281)		(n = 328)		(n = 294)		
	n	%	n	%	n	%	n	%	P^2^
**Overall supplement use**	586	64.9	163	58.0	204	62.2	219	74.5	0.0001
**Specific supplement use** 3									
Folic acid	406	45.0	141	50.2	144	43.9	121	41.2	0.07
Iron	380	42.1	52	18.5	140	42.7	188	63.9	<0.0001
Magnesium	289	32.0	57	20.3	111	33.8	121	41.2	<0.0001
Vitamin B6	240	26.6	48	17.1	105	32.0	87	29.6	<0.0001
Thiamin	233	25.8	49	17.4	105	32.0	79	26.9	0.0002
Riboflavin	229	25.4	46	16.4	103	31.4	80	27.2	<0.0001
Vitamin E	205	22.7	39	13.9	95	29.0	71	24.1	<0.0001
Vitamin B12	197	21.8	46	16.4	83	25.3	68	23.1	0.02
Zinc	187	20.7	41	14.6	83	25.3	63	21.4	0.004
Vitamin B8	182	20.2	38	13.5	79	24.1	65	22.1	0.004
Iodine	182	20.2	37	13.2	84	25.6	61	20.7	0.0007
Pantothenic acid	165	18.3	42	14.9	70	21.3	53	18.0	0.07
Other minerals^4^	148	16.4	31	11.0	64	19.5	53	18.0	0.01
Vitamin D	140	15.5	35	12.5	60	18.3	45	15.3	0.1
Vitamin C	142	15.7	23	8.2	68	20.7	51	17.3	0.0001
Niacin	134	14.8	37	13.2	51	15.5	46	15.6	0.6
ω3 fatty acids	99	11.0	20	7.1	47	14.3	32	10.9	0.02
Calcium	92	10.2	17	6.0	36	11.0	39	13.3	0.02
Other herbal supplement	72	8.0	20	7.1	26	7.9	26	8.8	0.97
Selenium	69	7.6	8	2.8	33	10.1	28	9.5	0.003
Retinol	41	4.5	6	2.1	18	5.5	17	5.8	0.08
Phosphorus	27	3.0	8	2.8	10	3.0	9	3.1	0.98
Evening primrose, borage, or cod liver oil	18	2.0	4	1.4	11	3.4	3	1.0	0.1
Beta-carotene	11	1.2	1	0.4	5	1.5	5	1.7	0.3
Fluoride	11	1.2	3	1.1	3	0.9	5	1.7	0.7
Acerola, guarana or cranberry supplement	10	1.1	3	1.1	5	1.5	2	0.7	0.6
Vitamin K	6	0.7	2	0.7	3	0.9	1	0.3	0.7
Fiber	4	0.4	3	1.1	1	0.3	0	0.0	0.6
Ginseng	3	0.3	2	0.7	0	0.0	1	0.3	0.8
Amino acids/proteins	3	0.3	1	0.4	1	0.3	1	0.3	0.99
Phytoestrogens	2	0.2	2	0.7	0	0.0	0	0.0	0.99
Lutein	1	0.1	0	0.0	0	0.0	1	0.3	-

1 DS users were defined as the subjects who used dietary supplement(s) at least 3 days a week at the time of the DS questionnaire.

2 Comparison of overall and specific DS use among pregnant women according to the trimester of pregnancy by unconditional logistic regression analysis adjusted for age.

3 Nutrients and other substances were consumed alone or in combination in the same DS.

4 Potassium, copper, lithium, manganese, chromium, and others.

### Circumstances of dietary supplement purchase

37 women were excluded from these analyses because of missing data. A very high proportion of pregnant women (86.7%) reported taking DS with a medical prescription or following physician advice (Table 3). The proportion of users of prescribed DS increased significantly from 67% in the 1^st^ trimester to 76% in the 3^rd^ trimester. 18.6% of pregnant women reported taking their supplements following advice of a pharmacist.

**Table 3:** Motives for dietary supplement purchase in pregnant women of the NutriNet-Santé cohort study.

	Pregnant women who	
Motives for dietary supplement purchase^2^	used supplements^1^	
	n	%
With medical prescription or advice	476	86.7
With medical prescription	382	69.6
Following medical advice	160	29.1
Following advice of a pharmacist	102	18.6
Following advice of a dietitian	6	1.1
Following advice of another health professional	31	5.6
Following advice of a friend/family member	45	8.2
Discovered DS in the store by themselves	25	4.6
Read about the DS in a book	17	3.1
Learned about the DS from the media (television, magazine, etc.)	17	3.1
Following advice received in the store (except in a pharmacy)	7	1.3
Saw an advertisement	2	0.4
Other circumstances	24	4.4
Do not know	1	0.2

1 DS users were defined as the subjects who used dietary supplement(s) at least 3 days a week at the time of the dietary supplement questionnaire. Data regarding circumstances of dietary supplement use were available for 94% of supplement users (i.e. 549 women out of 586).

2 Several answers possible.

### Dietary intake associated with DS use

Among the 903 pregnant women included in this study, 74% (n = 666) provided dietary data during their pregnancy and were normo-reporters; thus they were included in the following analyses. Most of those participants (73%) provided three 24 h dietary records, 17% provided 2 dietary records and only 10% provided only 1 record. DS users had significantly higher dietary intakes of most vitamins and minerals (i.e. thiamin, riboflavin, vitamin B6, folic acid, beta-carotene, vitamin E, iron, magnesium and potassium) (Table 4). DS users had slightly lower intakes of vitamin D than did DS non-users. Regarding folic acid intake from food, only 181 (27%) pregnant women reached the recommended dose of 0.4 mg/day and this proportion was significantly higher in folic acid supplement users than in non-users (33% vs. 23%; P = 0.002, data not tabulated).

**Table 4:** Daily dietary nutrient intake of pregnant women of the NutriNet-Santé study, overall and according to dietary supplement use.

	All^1^		Non-Users of		Users of		
			supplements		supplements^2^		
	(n = 666)		(n = 237)		(n = 429)		
	mean	SD	mean	SD	mean	SD	P^3^
Energy (kcal)	1999.1	459.3	1978.5	463.5	2010.4	457.0	0.4
Alcohol (g)	0.2	1.7	0.2	1.8	0.2	1.6	0.6
Total carbohydrates (g)	222.1	58.0	218.5	59.7	224.2	57.0	0.2
Simple carbohydrates (g)	110.6	37.3	107.5	40.3	112.3	35.5	0.07
Starches (g)	110.9	34.3	110.3	34.3	111.2	34.4	0.6
Fiber (g)	19.6	6.6	18.8	6.3	20.0	6.8	0.09
Proteins (g)	79.6	19.2	79.1	18.3	80.0	19.7	0.8
Total lipids (g)	87.0	26.4	86.7	26.2	87.2	26.5	0.3
Saturated fatty acids (g)	37.7	12.9	38.0	12.9	37.6	12.9	0.07
Monounsaturated fatty acids (g)	31.9	10.6	31.4	10.4	32.2	10.7	0.8
All polyunsaturated fatty acids (g)	11.3	4.6	11.2	5.0	11.3	4.3	0.8
n-3 polyunsaturated fatty acids (g)	1.2	0.6	1.2	0.7	1.2	0.6	0.5
n-6 polyunsaturated fatty acids (g)	9.3	4.2	9.3	4.7	9.4	3.9	0.9
Thiamin (mg)	1.3	0.5	1.3	0.5	1.4	0.5	0.01
Riboflavin (mg)	1.8	0.7	1.8	0.6	1.9	0.7	0.01
Niacin (mg)	17.9	6.4	17.4	5.9	18.3	6.6	0.2
Pantothenic acid (mg)	5.5	1.7	5.4	1.7	5.6	1.7	0.3
Vitamin B6 (mg)	1.8	0.6	1.7	0.6	1.8	0.7	0.004
Folate (µg)	344.8	116.5	328.6	111.3	353.7	118.4	0.02
Vitamin B12 (µg)	4.7	5.7	5.1	8.6	4.5	3.1	0.2
Retinol (µg)	507.2	679.9	521.5	747.9	499.2	640.0	0.5
Beta carotene (µg)	3289.5	2562.0	2938.9	2006.5	3483.1	2805.9	0.02
Vitamin C (mg)	131.6	95.7	124.0	63.1	135.8	109.4	0.2
Vitamin D (µg)	2.5	1.9	2.7	2.3	2.4	1.5	0.03
Vitamin E (mg)	12.0	4.9	11.4	5.2	12.3	4.7	0.04
Sodium (mg)	2618.4	837.3	2584.6	760.7	2637.1	877.0	0.8
Calcium (mg)	1037.7	330.1	1008.9	327.0	1053.6	331.1	0.1
Iron (mg)	13.0	4.6	12.2	4.0	13.4	4.9	0.003
Magnesium (mg)	320.7	92.5	305.7	78.4	329.0	98.5	0.003
Phosphorus (mg)	1298.9	321.8	1278.7	304.7	1310.0	330.7	0.52
Potassium (mg)	3012.2	764.1	2905.3	728.4	3071.3	777.7	0.02
Zinc (mg)	10.6	3.2	10.4	2.9	10.7	3.4	0.6

1 In pregnant women who provided at least one dietary record during their pregnancy.

2 DS users were defined as the subjects who used dietary supplement(s) at least 3 days a week at the time of completion of the dietary supplement questionnaire.

3 Unconditional logistic regression analyses adjusted for age, number of 24 h records and energy intake.

## Discussion

The present study highlighted demographic, socioeconomic and lifestyle disparities associated with DS use. Users of DS in general and of folic acid in particular were slightly older and belonged to higher socioeconomic classes, consistent with reports from other developed countries [23], [24], [27], [28], [35], [36]. Demographic and socioeconomic disparities associated with nutritional behaviour during pregnancy are of major public health importance as they are the precursors of socioeconomic inequalities regarding the health status of the next generation [37]. While some medicinal supplements recommended during pregnancy were partly reimbursed by social security/assistance programmes, the extra cost to the patient may deter DS purchases in low-income households. Besides, women with low income likely visit physicians less often and are might be less aware of nutritional recommendations compared to their more affluent counterparts.

The fact that women who already had children took fewer DS in general and folic acid in particular is also of major concern and has been observed in other countries [4], [28], [36]. This may be related to the fact that women who have already been pregnant in the past have fewer physician consultations in early pregnancy and/or are less compliant with the physician's recommendations.

Disparities between pregnant DS users and non-users also appeared as regards nutritional intake from food. Indeed, DS users had significantly higher dietary intakes of most vitamins and minerals, as previously reported in pregnant women [24] and in the general adult population [38]. A recent study in the Netherlands showed an inverse association between a Mediterranean diet rich in fruit, vegetables, fish, legumes and cereals and the risk of spina bifida in the offspring [39]. Thus, diet quality during pregnancy is of major public health importance. Our results highlight a combination of two risk factors (a poorer diet and an absence of supplementation for key nutrients) that may act synergically to increase the risk of disease in the foetus.

Whereas medical prescription or advice represented about 55% of DS use in the general NutriNet-Santé study [38], this proportion was much higher in pregnant women (86.7%), while self-medication with DS was still reported by about 15% of the pregnant women. This proportion was much lower than those documented in Australian and US studies [36], [40]. To our knowledge, such data have not been published for other European countries. In our study, 18.6% of DS users reported taking their supplements following advice of a pharmacist. It has been suggested that some pharmacists might be ill-equipped to counsel pregnant women about these products, and an ethical issue stemming from the profit-motive may occur [41].

During pregnancy, the nutritional requirements for several key nutrients (folic acid, iron, vitamin D and iodine in particular) increase, hence, supplemental intake under medical supervision may be beneficial. In our study, only 27% of pregnant women reached the recommended folate intake of 0.4 mg/day with food only. In turn, folic acid was the most frequently used nutrient in DS in our study. The proportion of folic acid users was higher than that observed in the 2010 French perinatal survey (40% took folic acid supplements during pregnancy and 24% before and/or at the time of conception [42]) but much lower than in other developed countries [4], [23], [36], [43], [44]. The potential harm of systematic folic acid supplementation has been questioned, but a recent meta-analysis of 13 trials showed that folic acid supplementation did not increase cancer risk at any site [45]. One of the issues regarding folic acid supplementation is that unplanned pregnancies possibly miss the critical period during which supplementation would be beneficial. [10], [46]

Iron requirements increase during pregnancy, especially over the last trimester [2], [12]. The current recommendation is to prescribe iron supplementation if women are at risk of insufficiency [16]. This is consistent with our results: iron was the second most frequently used supplemental nutrient, and its use tripled between the first and the last trimester, reaching 64% of users. However, this proportion is lower than in other developed countries [23], [28], [47].

Next, the current practice in France is to prescribe a single dose of 100 000 IU of vitamin D at the sixth month of pregnancy notably when the last trimester would take place in the winter [16]. However, our DS questionnaire (designed for the general population) did not capture information about the use of single-dose vitamin D medication. Nonetheless, our study provides important data on regular vitamin D supplement use, which concerned 15.5% of pregnant women.

The World Health Organization recommends a dose of 250 µg/d of iodine for pregnant women if access to iodized salt cannot be guaranteed [48]. In France, no systematic iodine supplementation is practiced [16]. In our study, the proportion of pregnant women using iodine supplementation reached 25.6% among those who were in their second trimester of pregnancy, as also observed in the US [15].

In contrast, several arguments encourage caution regarding supplement use as self-medication during pregnancy. They pertain to the potential toxicity associated with overdose of some nutrients or bioactive compounds and the potential deleterious effects of some herbal supplements, alone or when combined with certain medications [40], [49]. Excessive retinol intake is associated with increased risk of teratogenicity [17], [50] and retinol supplementation during pregnancy is not recommended. In our study, 5.8% of pregnant women used retinol supplements during the third trimester. A recent study showed that high maternal dietary and supplemental intake of vitamin E (>14.9 mg/day) was associated with a nine-fold increased risk of coronary heart disease in the offspring [18]. Thus, vitamin E DS use during pregnancy is of major concern. In our study, mean intake of vitamin E from food was 12 mg/day and nearly 30% of women used vitamin E supplements during the second trimester of pregnancy. 11% of the pregnant women used herbal supplements and only two women in this study took phyto-oestrogen supplementation.

To our knowledge, this epidemiologic study is the first which examined DS use and its correlates in French pregnant women. Its strengths include a substantial number of subjects, detailed evaluation of socioeconomic and lifestyle factors associated with DS use and detailed information on dietary intake.

Several limitations should be acknowledged. Caution is needed when extrapolating our results to pregnant women in general as this study was based on a sample of volunteers involved in an Internet-based cohort study on nutrition and health. Compared to pregnant women in the general French population, those participating in our study were older, better educated, and belonged to higher socioprofessional categories [42]. However, the direction of bias is predictable and suggests that supplement use in French pregnant women is probably slightly lower than what is observed in the present study. In addition, the major objective of this work was to investigate the associations between DS use and several individual-level correlates. Thus, the diversity of the sample (rather than its representativeness) is regarded the important parameter. Besides, this study included about 8% of women from lower socioprofessional categories (a population group that is usually difficult to reach), allowing us to perform between-class comparisons. Second, no information was available regarding folic acid and other DS use before pregnancy, while this period is critical regarding NTD prevention. Third, the distinction between medically-assisted versus spontaneous and planned versus unplanned pregnancies could not be made in this study while these factors may influence DS use [51]. Fourth, longitudinal follow-up of DS use during pregnancy was not available in this study (women were divided according to trimester groups in a cross-sectional manner). Fifth, although three dietary records are appropriate to adequately estimate energy intake [52], they may not capture all of the variability of dietary intake during the entire pregnancy. Next, the nutrient doses from the DS were not quantified. Finally, no information was available regarding biomarkers of nutritional status or ethnicity in this study, since a specific authorization is required in France to collect such sensitive data.

## Conclusion

This study provides new and detailed information on DS use and its correlates during pregnancy, highlighting socioeconomic differences in that dietary behaviour. In particular, women from lower socioeconomic classes were less likely to benefit from folic acid supplementation. 15% of pregnant women relied on self-medication. Even in this relatively well-educated and well-off population, folic acid supplementation at the beginning of pregnancy remained insufficient (only 50%), whereas only 27% of women reached the recommended 0.4 mg/day with food intake. In contrast, irrelevant supplementation practices have been identified (notably for retinol and vitamin E). It appears necessary to increase awareness among health professionals regarding the importance of recommending use of the right nutrient at the right moment (not only for the first but also for subsequent pregnancies; pre- and post-conception), while avoiding unnecessary (and even potentially hazardous) supplementation, with special attention paid to the lower socioeconomic strata.
